# Primary hemophagocytic lymphohistiocytosis in adults: the utility of family surveys in a single-center study from China

**DOI:** 10.1186/s13023-017-0753-7

**Published:** 2018-01-22

**Authors:** Zhili Jin, Yini Wang, Jingshi Wang, Jia Zhang, Lin Wu, Zhuo Gao, Wenyuan Lai, Zhao Wang

**Affiliations:** Department of Hematology, Beijing Friendship Hospital, Capital Medical University, 95 Yong An Road, Xicheng District, Beijing, 100050 China

**Keywords:** Primary hemophagocytic lymphohistiocytosis in adults, Family surveys, Immunological markers, Allogeneic hematopoietic stem cell transplantation

## Abstract

**Background:**

This study investigated the clinical characteristics of primary hemophagocytic lymphohistiocytosis (HLH) in adults, including immunological markers, pedigree findings, and conditions of allogeneic hematopoietic stem cell transplantation (Allo-HSCT).

**Methods:**

The study included clinical data of 18 adult patients with primary HLH treated in our center from June 2010 to January 2017.

**Results:**

Of these 18 cases, pathogenic variants were found in the following genes: *PRF1* (*n* = 11), *UNC13D* (*n* = 5), *SH2D1A* (*n* = 2), *RAB27a* (n = 1), and *LYST* (n = 2). One patient had pathogenic variants in both *PRF1* and *UNC13D* genes, one patient had pathogenic variants in both *LYST* and *UNC13D* genes and another patient had pathogenic variants in both *PRF1* and *SH2D1A* genes. Additionally, 3 of the 18 cases involved homozygous pathogenic variants, while 2 cases involved hemizygous pathogenic variants. The remaining 13 cases involved compound heterozygous pathogenic variants. The natural killer (NK) cell activity test was conducted in all 18 cases where 14(77.8%)patients showed reduction in NK cell activity. Furthermore, this article presents 3 representative results of the pedigree findings from 12 patients who underwent family surveys. The 8 patients who underwent Allo-HSCT had a median survival of 27.2 months, as compared with the median survival of 7 months for the10 patients who did not undergo Allo-HSCT, a significant difference between the two groups of patients (*p* = 0.006).

**Conclusion:**

*PRF1* was one of the most commonly mutated gene in adult patients with primary HLH. Family surveys and immunological markers were important for the HLH diagnosis and the selection of an appropriate donor. Allo-HSCT was an effective therapy for adult primary HLH.

## Background

Primary HLH is an autosomal and/or X-linked recessive inheritance characterized by immune disorders. In the past, diagnosis of patients with primary HLH was based on a disease onset at an early age and a positive family history. In 1999, a research group in Paris reported the first primary HLH-related gene map [1]. The “HLH-2004: diagnostic criteria for hemophagocytic lymphohistiocytosis” proposed by the Histiocyte Society clearly reported that genetic defects are the gold standard of primary HLH. At least 12 relevant genes associated with primary HLH have been reported, include: *PRF1, UNC13D, STX11, STXBP2, SH2D1A, BIRC4, RAB27A, LYST, ADTB3A, ITK, CD27* and *MAGT1* [2]. With the gradual understanding of adult primary HLH, age is no longer the basis for the diagnosis. Currently, delayed onset of adult primary HLH has been considered to relate to the gene pathogenic variants, the pathogenic variant patterns, and the presence of a trigger.

Immunological markers are important for the early diagnosis of primary HLH. Janka et al. [3] showed that NK cell activity was reduced in almost all patients at the early stage of primary HLH. Thus, timely detection of NK cell activity is important for the early diagnosis of the disease. CD107a detection can be used as a highly sensitive method for the identification of primary HLH [4]. Timely detection of the immunological markers has been considered to be significantly better than genetic testing and has been used as an effective and rapid screening for primary HLH in the diagnostic processes of international studies [4, 5].

Early diagnosis of primary HLH may require a positive family history gained from family surveys of individuals with abnormal immunological markers or suspected primary HLH as a supportive basis to help confirm the diagnosis. Carrier testing of family members through gene sequencing, cytotoxicity analysis and protein measurements can result not only in the identification of yet healthy homozygous carriers but also aid the selection of suitable donors in the family.

The long-term strategy of HLH treatment is to correct the immunodeficiency, so patients with primary HLH require Allo-HSCT to correct the potential genetic defects. Allo-HSCT is recommended by HLH-94 for FHL and refractory recurrent HLH patients, using the traditional myeloablative pretreatment regimen [6]. Combined chemotherapy only temporarily controls the incidence of FHL effectively. Patients who do not receive Allo-HSCT cannot achieve long-term survival [7]. Henter et al. [8] reported the first multicenter prospective study in 2002 and showed that remission occurred in 119 primary HLH patients after combined chemotherapy and subsequent HSCT. Clear recovery was observed with an overall survival rate of 55% in the 43-year follow up where the majority of deaths occurred after the early diagnosis or before the transplantation. More and more clinical studies have shown that Allo-HSCT is the only curative regimen for adult primary HLH. Currently, publications regarding adult primary HLH diagnosis and treatment are few and most of them are case reports. We conducted a related study to identify the clinical characteristics in the adult patients with primary HLH.

## Methods

### Research subject

Eighteen adult patients with primary HLH, diagnosed and treated in Beijing Friendship Hospital, Capital Medical University from June 2010 to January 2017, were included in this study. All patients (≥ 18 years old) underwent genetic testing and were found to have primary HLH-related genetic defects. (Supplement: Zhang K et al. considered that “In patients with combination defects involving 2 genes in the degranulation pathway, CD107a degranulation was decreased, comparable to patients with biallelic mutations in one of the genes in the degranulation pathway. This suggests a potential digenic mode of inheritance of FHL as a result of a synergistic function effect within genes involved in cytotoxic lymphocyte degranulation.” [9] and Sepulveda et al. considered that an additive effect of HLH mutations in different genes has been shown in mouse models [10]. So the patient who has defects involving 2 genes in the degranulation pathway and CD107a degranulation decreased (P14) and the patient who has polygenic inheritance additive effect(P15) were diagnosed with presumably primary HLH in our center.

### Immunological markers and family surveys of adult HLH patients

Immunological markers of the 18 adult patients with primary HLH were assessed using a cytotoxicity assay, CD107a degranulation assay, perforin, granzyme, signaling lymphocytic activation molecule (*SLAM*)-associated protein(*SAP*), and x-linked inhibitor of apoptosis protein (*XIAP*) detections. Gene sequencing of family members of the patients were performed. Cytotoxicity and involved protein expression of some family members were detected.

### Gene sequencing

Using specific-primer design and PCR on DNA extracted from mononuclear cells, the exon and related cleavage products of HLH-related genes were obtained. This was followed by bi-directional Sanger sequencing.

The pathogenic variants and types were identified by Esembl genomic databases. To see if a pathogenic variant has been previously reported before, some information were identified by the Exome Aggregation Consortium(ExAC)and Human Gene Mutation Database(HGMD). The pathogenicity of single nucleotide polymorphisms was predicted by Polyphen2 and SIFT databases. Polyphen2 > 0.95 or SIFT < 0.05 predicted that the amino acid change caused by pathogenic variant may affect protein expression or function.

### HSCT

Pre-transplant treatment was conducted based on an HLH-94/04 regimen (i.e., etoposide, glucocorticoid, and cyclosporine) and a DEP regimen (i.e., methylprednisolone, liposomal adriamycin, and etoposide). For donor selection of the HSCT, complete matching and gene screening of patients’ parents and siblings were conducted, followed by complete family surveys. NK cell activity and CD107a detections, as well as primary hemophagocytic gene screening, were performed in all donors to exclude the possibility of primary hemophagocytosis.

### Statistical methods

SPSS version 22.0 was used for statistical analysis. All the data that conformed to the normal distribution was represented by x±SD and paired t test was used, while non-normal-distributed data was presented using the median and extreme values, Wilcoxon’s rank-sum test was used. Statistical difference was defined as *P* < 0.05, significant statistical difference was defined as *P* < 0.01.

## Results

### General conditions of adult patients with primary HLH

Of the 18 adult HLH patients, there were 10 males(55.6%)and 8 females(44.4%), with the median age of onset at 25.5 years (18, 54). Of these 18 cases, pathogenic variants were found in the following genes: *PRF1* (*n* = 11), *UNC13D* (*n* = 5), *SH2D1A* (*n* = 2), *RAB27a* (n = 1), and *LYST* (n = 2). One patient had pathogenic variants in both *PRF1* and *UNC13D* genes, one patient had pathogenic variants in both *LYST* and *UNC13D* genes and another patient had pathogenic variants in both *PRF1* and *SH2D1A* genes. Three of the 18 cases involved homozygous pathogenic variants, while 2 cases involved hemizygous pathogenic variants. The remaining 13 cases involved compound heterozygous pathogenic variants. Pathogenic variants patterns included missense pathogenic variant, nonsense pathogenic variant, and frameshift pathogenic variant. Adult primary HLH gene pathogenic variants are listed in Table 1.

**Table 1:** Adult primary HLH gene pathogenic variants

patient number	age at diagnosis	gender	involved gene	site and type of pathogenic variants	change in amino acid	NM_	Polyphen2(HDIV)	SIFT	ExAC_ALL	HGMD (Disease)
P01	18	F	PRF1	c.1168C > T (heterozygous nonsense pathogenic variant)	p. R390X	NM_001083116 NM_005041	–	–	0.000008654	Cytophagic histiocytic panniculitis
				c.1349C > T (heterozygous missense pathogenic variant)	p.T450 M		1	0.001	0.00004957	Haemophagocytic lymphohistiocytosis, familial
P02	19	M	PRF1	c.1349C > T (homozygous missense pathogenic variant)	p.T450 M	NM_001083116 NM_005041	1	0.001	0.00004957	Haemophagocytic lymphohistiocytosis, familial
P03	18	M	PRF1	c.172 T > C (heterozygous missense pathogenic variant)	p. S58P	NM_001083116 NM_005041	0.159	0.055	–	–
				c.1083_1094del(heterozygou s non-frameshift pathogenic variant)	p.361_365del		–	–	–	–
P04	54	F	PRF1	c.65delC (heterozygous frameshift pathogenic variant)	p. P22RfsX29	NM_001083116 NM_005041	–	–	0.0000108	Haemophagocytic lymphohistiocytosis, familial
				c.674G > A (heterozygous missense pathogenic variant)	p.R225Q		0.05	0.366	0.0001	–
P05	27	M	PRF1	c.503G > A (heterozygous missense pathogenic variant)	p. S168 N	NM_001083116 NM_005041	0.035	0.45	0.00002473	Haemophagocytic lymphohistiocytosis, familial
				c.65delC (heterozygous missense pathogenic variant)	p.P22RfsX29		–	–	0.0000108	Haemophagocytic lymphohistiocytosis, familial
P06	18	F	PRF1	c.1090_109ldel(heterozygous frameshift pathogenic variant)	p. T364fsX93	NM_001083116 NM_005041	–	–	0.000008701	Haemophagocytic lymphohistiocytosis, familial
				c.1349C > T (heterozygous missense pathogenic variant)	p.T450 M		1	0.001	0.00004957	Haemophagocytic lymphohistiocytosis, familial
P07	18	M	PRF1	c.65delC (heterozygous frameshift pathogenic variant)	p. P22RfsX29	NM_001083116 NM_005041	–	–	0.0000108	Haemophagocytic lymphohistiocytosis, familial
				c.1349C > T (heterozygous missense pathogenic variant)	p.T450 M		1	0.001	0.00004957	Haemophagocytic lymphohistiocytosis, familial
P08	18	F	PRF1	c.380A > G (heterozygous missense pathogenic variant)	p. N127S	NM_001083116 NM_005041	0.999	0	–	–
				c.853_855delAAG(heterozygous frameshift pathogenic variant)	p.K285del		–	–	0.00005766	Haemophagocytic lymphohistiocytosis, familial
P09	18	F	PRF1	c.46C > T (heterozygous missense pathogenic variant)	p. P16S	NM_001083116 NM_005041	0.995	0.19	–	–
				c.1066C > T (heterozygous missense pathogenic variant)	p.R356W		0.208	0.016	0.000008576	Haemophagocytic lymphohistiocytosis, familial
P10	36	M	PRF1	c.133G > A (heterozygous missense pathogenic variant)	p. G45R	NM_001083116 NM_005041	1	0	–	Haemophagocytic lymphohistiocytosis, familial
				c.1228C > T (heterozygous missense pathogenic variant)	p. R410W		1	0.009	0.00005111	Haemophagocytic lymphohistiocytosis, familial
			UNC1 3D	c. 1280G > A (heterozygous missense pathogenic variant)	p.R427Q	NM_199242	1	0.005	0.00008298	–
P11	52	M	UNC13D	c.2588G > A (homozygous missense pathogenic variant)	p.G863D	NM_199242	1	0	0.0004	Haemophagocytic lymphohistiocytosis, familial
P12	24	F	UNC13D	c.407G > A (heterozygous missense pathogenic variant)	p.C136Y	NM_199242	1	0	–	–
				c.640C > T (heterozygous nonsense pathogenic variant)	p.R214X		–	–	0.00000824	Haemophagocytic lymphohistiocytosis, familial
P13	28	M	UNC13D	c.3134C > T (heterozygous missense pathogenic variant)	p. T1045 M	NM_199242	0.598	0.158	0	–
				c.2553 + 5C > G (heterozygous missense pathogenic variant)	–		–	–	0.0008	Haemophagocytic lymphohistiocytosis, familial
P14	18	M	UNC13D	c.1120C > A(heterozygous missense pathogenic variant)	p.P374T	NM_199242	0.96	0.22	–	–
			LYST	c.11268-5 T > −(heterozygous missense pathogenic variant)	–	NM_000081	–	–	–	–
P15	27	M	PRF1	c.127C > A (heterozygous missense pathogenic variant)	p. L43 M	NM_001083116 NM_005041	0.926	0.099	–	–
			SH2D 1A	c.7G > T(hemizygous missense pathogenic variant)	p.A3S	NM_001114937 NM_002351	0	0.84	0.0002	Lymphoproliferative syndrome, X-linked
P16	32	M	SH2D1A	c.32 T > G (hemizygous missense pathogenic variant)	p.I11S	NM_001114937 NM_002351	0.999	0.002	–	–
P17	34	F	RAB27a	c.244C > T (homozygous missense pathogenic variant)	p.R82C	NM_004580 NM_183234 NM_183235 NM_183236	1	0	0.000008303	Immunodeficiency
P18	32	F	LYST	c.8368A > C (heterozygous missense pathogenic variant)	p. K2790Q	NM_000081 NM_001301365	0.38	0.021	0.0008	–
				c.11268-4A > T (heterozygous missense pathogenic variant)			–	–	–	–

### Immunological marker and family surveys of adult patients with primary HLH

#### Immunological marker of adult patients with primary HLH

Detection of immunological markers, including cytotoxicity, CD107a, perforin, granzyme, *SAP*, and *XIAP* were performed in the 18 primary HLH patients (Table 2). The NK cell activity test was conducted in all 18 cases where 14(77.8%)patients showed reduction in NK cell activity. The 8 patients who completed the CD107a degranulation assay, P08, P09, and P10 showed normal CD107a expression whereas P11, P12, P13, P14, and P17 showed reduced CD107a expression (In 6 patients with UNC13D or RAB27a or LYST pathogenic variants who also completed the CD107a degranulation assay, 5 patients have decreased CD107a expression, P10 showed normal CD107a expression) Among the 8 patients who completed the perforin assay, P03, P06, P08, and P09 had reduction of perforin levels, while P10, P11, P13, and P17 had normal perforin levels. (In 5 patients with PRF1 pathogenic variants who also completed the perforin assay, 4 patients have decreased perforin expression) No significant abnormality was noted among the 3 patients who completed the *SAP* and *XIAP* protein assays, and their gene detections showed no involvement of *SH2D1A* and *XIAP* gene.

**Table 2:** Immunological marker of adult patients with primary HLH

number	NK ≥15.11% normal	CD107a (ΔCD107a) > 10% normal	CD107a (ΔMFI) ≥2.8 normal	ΔPRF1 NK ≥ 81% CTL ≥ 2% normal	ΔGranzymeB NK ≥ 77% CTL ≥ 6% normal	ΔSAP NK (26–70) % CTL (43–87) % normal	XIAP NK (59–100) % CTL(61–100)% normal	classification
P01	18.68%	–	–	–	–	–	–	FHL2
P02	11.37%	–	–	–	–	–	–	FHL2
P03	10.74%	–	–	NK 6.93% CTL6.66%	NK 75% CTL 40%	–	–	FHL2
P04	1.74%	–	–	–	–	–	–	FHL2
P05	15.69%	–	–	–	–	–	–	FHL2
P06	9.9%	–	–	NK0.31% CTL0.16%	–	–	–	FHL2
P07	10.6%	–	–	–	–	–	–	FHL2
P08	16.20%	20.05%	3.0%	NK 78% CTL 51%	NK 97% CTL 94%	NK62.1% CTL57.69%	NK66.03% CTL90.96%	FHL2
P09	11.29%	13.75%	3.0%	NK 33% CTL 1%	NK 99.87% CTL 99.77%	–	–	FHL2
P10	9.86%	29.95%	3.7%	NK95.88% CTL49.19%	NK 97.02% CTL 95.80%	NK 86.88% CTL82.66%	NK 87.94% CTL 74.91%	FHL2
P11	13.5%	10.76%	2.5%	–	–	–	–	FHL3
P12	12.27%	5.52%	1.5%	NK89.36% CTL70.07%	NK77.35% CTL77.04%	–	–	FHL3
P13	17.59%	9.18%	5.9%	NK 85.1% CTL57.32%	NK 71.52% CTL55.00%	NK 65.92% CTL 69.03%	NK 95.52% CTL 97.61%	FHL3
P14	11.14%	1.36%	0.86%	–	–	–	–	–
P15	13.57%	–	–	–	–	–	–	XLP-1
P16	11.08%	–	–	–	–	–	–	XLP-1
P17	5.4%	9.36%	2.2%	NK 89.42% CTL4.26%	NK 89.48% CTL 5.92%	–	–	GS-2
P18	13.67%	–	–	–	–	–	–	CHS

#### Family surveys of adult patients with primary HLH

Among the 12 patients who underwent family surveys, some of their relatives completed gene sequencing and detection of immunological markers. This study was selected to further interpret the representative pedigree findings of 3 cases, including P11 (with *UNC13D* homozygous missense pathogenic variant), P03 (with *PRF1* compound heterozygous pathogenic variant), and P16 (with *SH2D1A* hemizygous missense pathogenic variant).

##### Investigation of families of individuals with homozygous pathogenic variants

**Fig. 1:**
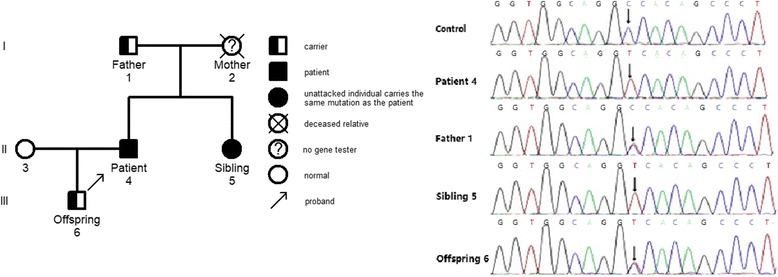
Family diagram and gene sequencing of P11

**Fig. 2:**
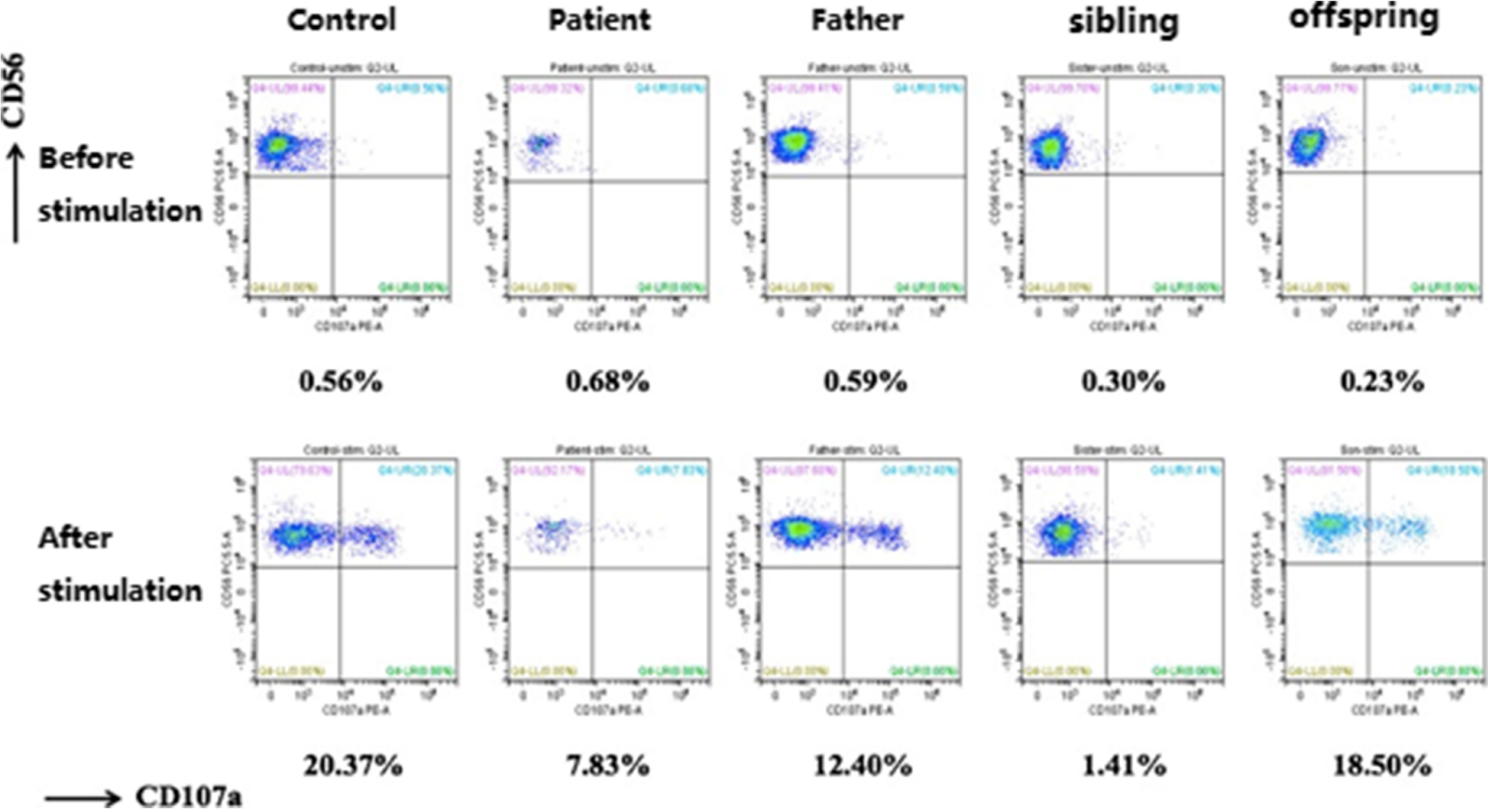
Representative image showing the CD107a expression

Investigation of the family of P11 showed that: proband, male, 52 years old. *UNC13D had a* homozygous missense pathogenic variant at c.2588G > A. Family surveys of P11 were confirmed by Sanger sequencing, which showed that the patient’s father and offspring also had heterozygous pathogenic variants. Patient’s mother did not complete the family survey. However, given the homozygous pathogenic variant of P11, patient’s parents should have heterozygous pathogenic variant at the same locus. In addition, P11’s sibling also had the same homozygous missense pathogenic variant but did not suffer from HLH. Cytotoxic degranulation (CD107a expression) of P11 and his sibling was reduced (Figs. 1 and 2).

##### Investigation of families of individuals with compound heterozygous pathogenic variants

**Fig. 3:**
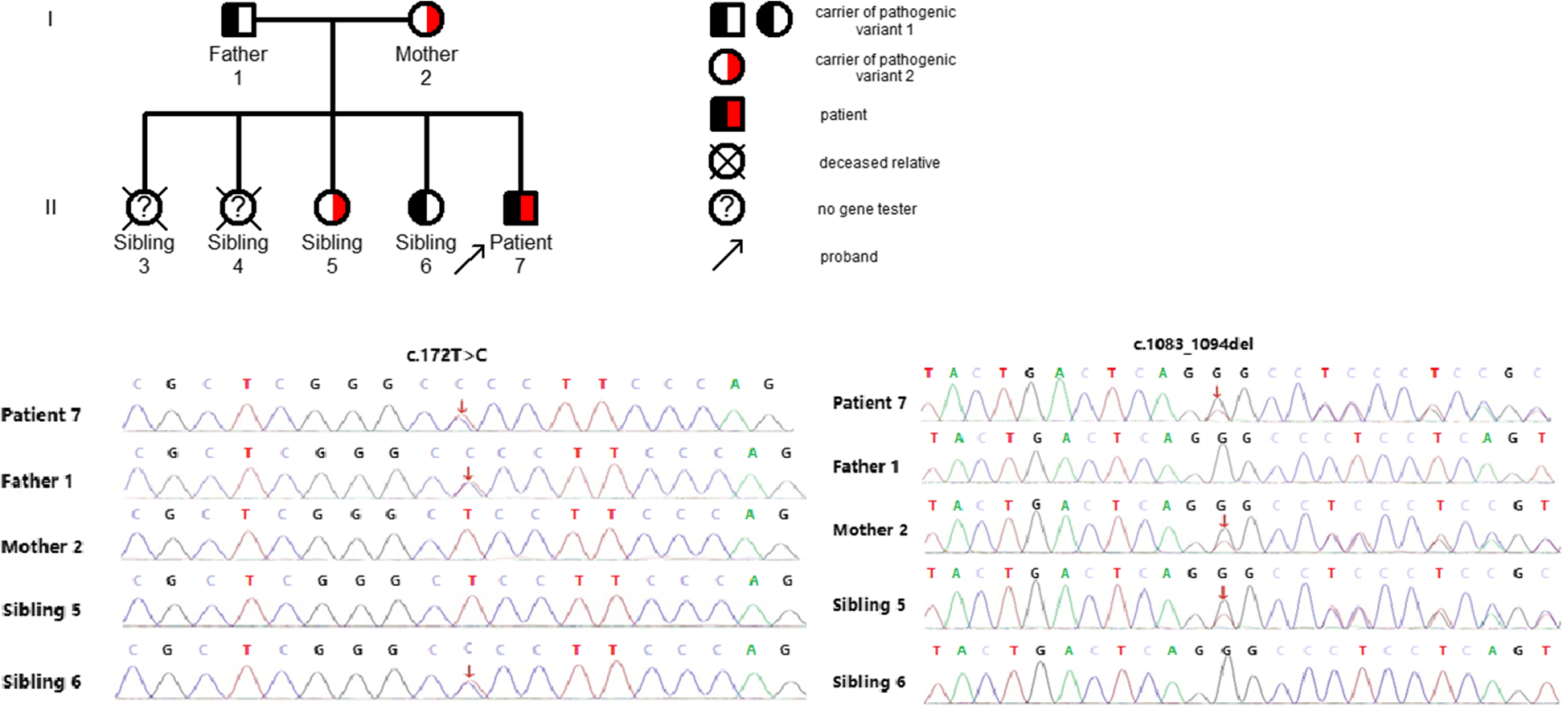
Family diagram and gene sequencing of P03

**Fig. 4:**
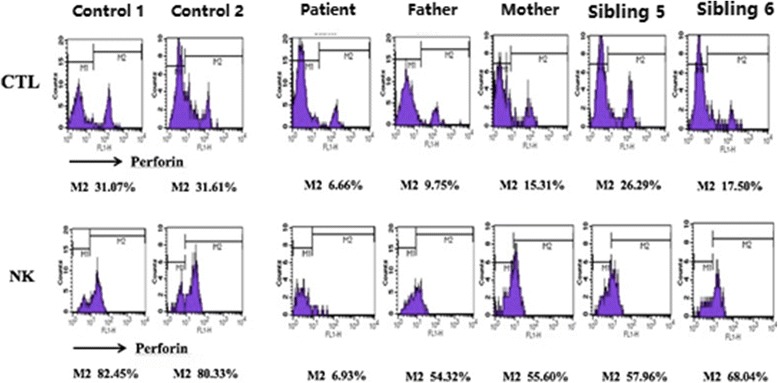
Representative image showing the perforin protein expression

Investigation of the family of P03 showed that: proband, male, 18 years old. *PRF1* had compound heterozygous pathogenic variants, including a heterozygous missense pathogenic variant at c.172 T > C and a non-frameshift pathogenic variant at c.1083_1094del. Family surveys of P03 showed that P03 had suspected a positive family history of HLH, as two elder siblings of P03 died from unexplained fevers in their childhoods. Through Sanger sequencing, the two pathogenic variants of P03 originated from P03’s parents. However, P03 also had two healthy siblings who carried different pathogenic variants (Figs. 3 and 4).

##### Investigation of families of individuals with Hemizygous pathogenic variants

**Fig. 5:**
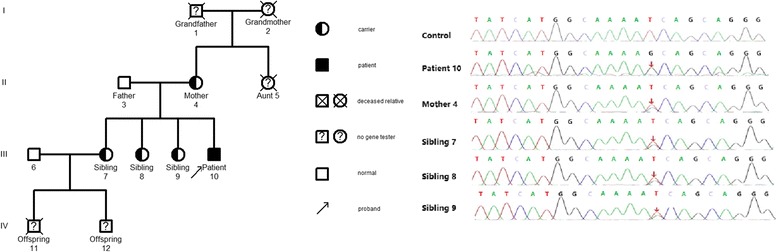
Family diagram and gene sequencing of P16

**Fig. 6:**
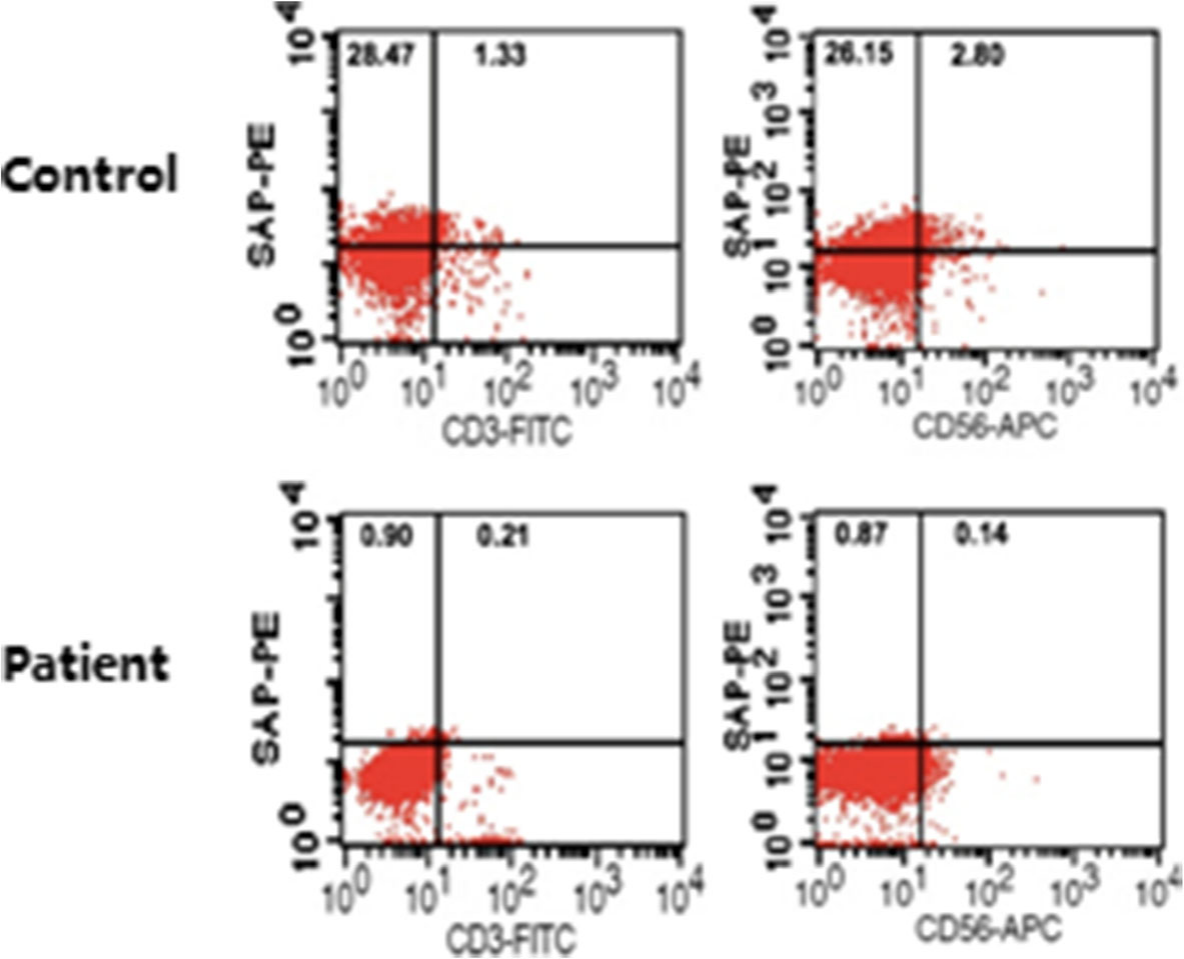
Representative image showing the SAP protein expression

Investigation of the family of P16 showed that: proband, male, 32 years old. *SH2D1A* had a hemizygous pathogenic variant at c.32 T > G, and the family surveys of P16 showed that P16’s maternal grandfather died at 30+ years old with an unknown cause of death. P16’s nephew (the offspring of P16’s elder sibling,1-year-old) simultaneously had the same onset with similar symptoms as P16 and died within a week after hospital admission. Through Sanger sequencing, the pathogenic variant of P16 originated from P16’s mother, and three siblings of P16 also carried the same pathogenic variant,P16’s mother and three siblings did not suffer from HLH. The expression of *SH2D1A* gene encoded relevant protein *SAP* of P16 was reduced (Figs. 5 and 6).

### Allo-HSCT in adult patients with primary HLH

Table 3 shows the general information of the patients with follow-ups until May 2017. Among the 8 patients who received Allo-HSCT, one HLH patient died while the remaining seven HLH patients survived. Of the 10 patients who did not receive Allo-HSCT, 7 patients died, 1 was lost to follow-up, the other 2 patients survived. The 8 patients who underwent Allo-HSCT had a median survival of 27.2 months, as compared with the median survival of 7 months for the10 patients who did not undergo Allo-HSCT, a significant difference between the two groups of patients (*p* = 0.006).

**Table 3:** The general information of adult patients with primary HLH who received Allo-HSCT

Patient’s number	P02	P03	P08	P11	P12	P14	P15	P17
donor origin	affinity	affinity	affinity	affinity	affinity	affinity	affinity	affinity
human leukocyte antigen (HLA) typing	5/10	5/10	10/10	5/10	10/10	5/10	5/10	5/10
stem cell type	PB	PB	PB	PB	PB	PB	PB	PB
pretreatment regimen	TBI/VP16/CTX	TBI/VP16 /Cy	TBI/VP16/Cy	TBI/VP16 /CTX	VP16/Bu/Cy	VP16/BU/FLU/ATG	TBI/VP-16/CTX	VP16/BU/ FLU/ATG
white blood cell viability (days)	+ 10	+ 11	+8	+ 12	+ 13	+ 10	+ 18	+ 15
platelet engraftment time (days)	+ 13	+ 12	+ 10	+ 13	+ 13	+ 15	+ 18	+ 15
+ 20 d donor chimerism rate	100%	99.6%	100%	100%	99.04%	100%	100%	97.78%
GVHD	III	–	–	I	–	–	–	–
pre-transplant EBV copy	0	0	5.1 × 10^4^	8.2 × 10^5^	0	1.5 × 10^4^	9.2 × 10^3^	0
post-transplant EBV copy	0	0	0	0	0	0	0	0
prognosis	survival	survival	survival	death	survival	survival	survival	survival

## Discussion

Primary HLH is a rapidly progressive and life-threatening disease characterized by a reduction of NK cell and cytotoxic T lymphocyte function caused by genetic defects, resulting in excessive immune activation. Patients with an onset before 2 years of age account for more than 90% of all patients [3]. At least 12 relevant genes associated with primary HLH have been reported, changes in gene sequences were reported in adult HLH patients in the subsequent studies. Delayed onset of adult primary HLH has been considered to relate to the gene pathogenic variants, the pathogenic variant patterns, and the presence of a trigger. For example, a study by Ueda et al. [11] analyzed the gene pathogenic variants in pediatric HLH patients and showed that nonsense and frameshift pathogenic variants that occurred in infants mostly happened in the classical onset of HLH while missense pathogenic variants occurred in the later onset of the disease at older ages. Pagel et al. [12] found that patients with *STXBP2* splice-site pathogenic variants, as compared with patients carrying nonsense pathogenic variants, had a higher age of onset with a median age of 4.1 years and 2 months, respectively. The age of onset of compound heterozygous pathogenic variants involved only the perforin or the degranulation pathway was later than the age of onset of the homozygous pathogenic variants [9]. The presence of a trigger also plays an important role in the incidence of primary HLH. For example, A91V is a milder class I perforin mutation with partial maturation and reduced but detectable perforin and NK function [13]. A91V confers genetic susceptibility for the development of FHL, but is not enough to trigger the disease on its own, the M. tuberculosis infection as synergistic factors play a role in the development of FHL [14]. In our data, in the family surveys of P11, the sibling of the proband P11 had the same homozygous pathogenic variant as P11. In addition, the cytotoxic degranulation (CD107a expression) was reduced in P11’s sibling but with no incidence of HLH. The plausible reason was that P11 had positive EBV-DNA(8.2 × 10^5^copies/ml)while P11’s sibling had negative EBV-DNA. In the family surveys of P16, the proband had positive EBV-DNA(8.8 × 10^4^copies/ml)detected when he suffered from HLH in 32 years old, P16’s maternal grandfather and nephew died with an unknown cause, we speculate that the possibility of the occurrence of HLH can not be excluded. P16’s mother and three siblings had no evidence of EBV infection or other triggering factor(s), although they carried the same pathogenic variant, they did not suffer from HLH. This would suggest that in addition to inherent immune deficiency, other triggering factor(s) might be involved in the incidence of primary HLH. Age was no longer the diagnostic basis of primary HLH. Our data also showed that approximately 72% of the 18 adult patients with primary HLH had compound heterozygous pathogenic variants. Some of the pathogenic variants(such as c.1349C > T)were reported before in other patients with late onset HLH [11], which also partly explained the reason for the HLH onset in adulthood.

Janka et al. [3] showed that NK cell activity was reduced in almost all patients at the early stage of primary HLH. Our result showed that the NK cell activity test was conducted in all 18 cases where 14(77.8%)patients showed reduction in NK cell activity. Thus, timely detection of NK cell activity is important for the early diagnosis of the disease. In this study, 4 primary HLH patients had normal NK cell activity, indicating that normal NK cell activity could not completely exclude the possibility of primary HLH as a diagnosis. Rapid immunological assays such as CD107a expression could be used to verify the HLH diagnosis and provide the etiology data that quickly divides HLH into two categories. Genetic defects, which affected the cytotoxic degranulation routes, led to the functional impairment of the transfer of lysosomal-related membrane glycoprotein CD107a to the cell surface, thereby significantly reducing the CD107a expression. NK degranulation analyses clearly distinguished the patients with granule exocytosis dysfunction, secondary HLH, or other hereditary defects such as *SAP* and *XIAP* deficiencies. A previous study has shown that patients with secondary HLH and other types of primary HLH have normal CD107a expression [4]. In this study, of the 8 patients who completed the CD107a detection, 3 of them showed normal CD107a expression. Five of the 8 patients had reduced CD107a expression, with *UNC13D* and *RAB27a* genes affected, which influenced the degranulation function. P10 with single heterozygous missense pathogenic variant of *UNC13D* showed normal CD107a expression, the reason may that the single heterozygous missense pathogenic variant of *UNC13D* was not enough to cause CD107a expression reduction. No significant abnormality was noted among the 3 patients who completed the *SAP* and *XIAP* protein assays, and their gene detections showed no involvement of *SH2D1A* and *XIAP* gene. These findings suggest that immunological marker detections and genetic testing results match each other well. However, in terms of timeliness,the detection of immunological markers was significantly superior to the genetic testing. Therefore, immunological markers played an important role in the early diagnosis of primary HLH.

Active HLH disease progresses rapidly, and without timely and effective treatment, the median survival time is only 2 months [15]. Allo-HSCT is a necessary means for the correction of immunodeficiency in primary HLH. Myeloablative stem cell transplantation(MAC)has a relatively high transplant-related mortality (30 to 50%). *XIAP*-mutant patients have less tolerance to bone marrow transplantation, suggesting the necessity of a lower toxicity in the regimens [16]. A recent study has suggested that a reduced-intensity conditioning (RIC) in alemtuzumab, fludarabine, and melphalan-based pretreatment reduces the toxicity and improves the survival of HLH patients. The three-year survivals of RIC and MAC groups in that particular study were 92% and 43%, respectively [17]. In this study, the two major pretreatment regimens included total body irradiation (TBI)/VP-16/cyclo-phosphamide, (CY)/antithymocyte globulin (ATG), and VP16/busulfan (BU)/fludarabine (FLU)/ATG. Jordan et al. believed that HLH patients had potential hepatic and pulmonary damage [18]. These patients, who received BU-based pretreatment regimens, also had high incidences of veno-occlusive disease (VOD) and pneumonia. Application of BU in the patients receiving Mylotarg or radiotherapy to the liver should be performed with high caution. Thus, this study applied the TBI/VP-16/CY/ATG pretreatment regimen in the primary HLH patients with liver damage. With regard to the timing of Allo-HSCT, a large-scale clinical trial proposed that once a primary HLH diagnosis is confirmed, the patient should receive HSCT as soon as possible [18]. Efficacy of the transplantation is closely related to the disease state prior to the transplantation. HSCT conducted after confirming HLH family history but before the presence of symptoms in the systems or HSCT conducted without HLH family history but with achieved clinical alleviation after drug treatment, can result in relatively high overall survival [19]. In this study, among the 8 HLH patients who received Allo-HSCT, all underwent a HLH-94/04 regimen and DEP regimen to achieve complete response or partial response afterwards. For the selection of transplant donors, a clinical trial has confirmed that the patients without a matching HLA donor can receive a transplant from haploid donors [20]. The results of commensurate or unrelated transplantation are comparable [17]. Relative donors should be chosen from the patients’ siblings who are without any known HLH genetic defect. However, assessment on the possibility of potential incidence of HLH using the family gene surveys may help the selection of commensurate donors. NK cell activity assay, CD107a detection, and primary hemophagocytic gene screening could also exclude the possibility of primary hemophagocytosis. Family surveys and the detection of immunological markers in adult patients with primary HLH strongly influenced the disease diagnosis and the selection of an appropriate transplant donor.

Allo-HSCT is the only curative regimen for primary HLH. Current reports in adult primary HLH treatment using Allo-HSCT are limited. Among the 8 adult primary HLH patients who received Allo-HSCT in this study, their chimerism after transplantation was complete. The 4 EBV-positive patients (P08,P11,P14,P15)in this study became EBV-negative after the Allo-HSCT, indicating that Allo-HSCT corrected the immunodeficiency and allowed the patients to have the ability to clear EBV. The median survival time of the Allo-HSCT group was 27.2 months, which was significantly higher than the non-Allo-HSCT group (*p* = 0.006). Allo-HSCT was an effective therapy for adult primary HLH.

## Conclusions

Untreated primary HLH patients have a very low survival rate. Family surveys and immunological markers were important for the HLH diagnosis and the selection of an appropriate donor. Allo-HSCT was an effective therapy for adult primary HLH. To date, the diagnostic and treatment data of adult primary HLH patients have been rarely reported. Therefore, we should better review existing cases, to identify the suitable timing to apply Allo-HSCT, select the appropriate transplant donor, and develop a more unified transplantation program to improve the treatment efficacy.

## Abbreviations

Allo-HSCT: Allogeneic hematopoietic stem cell transplantation
ATG: Antithymocyte globulin
BU: Busulfan
CY: Cyclo-phosphamide
ExAC: Exome Aggregation Consortium
FLU: Fludarabine
HGMD: Human Gene Mutation Database
HLH: Hemophagocytic lymphohistiocytosis
MAC: Myeloablative stem cell transplantation
NK: Natural Killer
RIC: Reduced-intensity conditioning
*SAP*: *SLAM*-associated protein
*SLAM*: Signaling lymphocytic activation molecule
TBI: Total body irradiation
VOD: Veno-occlusive disease
*XIAP*: X-linked inhibitor of apoptosis protein
